# Changes in the Faculty of the Baltimore College of Dental Surgery

**Published:** 1869-03

**Authors:** 


*Changes in the Faculty of the Baltimore College of Dental Surgery.*—The chair of Anatomy, made vacant by the resignation of Professor Russell Murdoch, has been filled by the appointment of E. Lloyd Howard, M.D. Professor of Anatomy. Professor Howard very acceptably performed the duties of Lecturer of Anatomy during the session now about to close.

Russell Murdoch, M.D. formerly Professor of Anatomy, has been appointed to the chair of Chemistry, made vacant by the death of Professor A. Snowden Piggot.

